# Some Practical Aspects of Bovine Tuberculosis

**Published:** 1895-11-09

**Authors:** 


An Institutional Journal of

The Medical Sciences and Hospital Administration, with

AN EXTRA NURSING SUPPLEMENT.

Vol. XIX.—No. 476.] [Nov. 9, 1895.

### Some Practical Aspects of Bovine Tuberculosis.

In matters agricultural the French politician is sometimes unpractical; but he is mostly both scientific and logical. In questions of the same order the English agriculturist is, as a rule, neither scientific, logical, nor practical. Tuberculosis in cattle is a highly important question from the point of view alike of the farmer's pocket and the public health. The French Minister of Agriculture has recognised this fact, and has determined to stamp out the disease at all costs. In our own country little or nothing has yet been done, either by way of prevention or extirpation.

To our thinking the French Minister, though sound in his principles, is as unnecessarily drastic in his measures as the Englishman is unscientifically dull. Let us try to put the whole case in a nut-shell. In the first place, the percentage of dangerously tuberculous cattle is comparatively small. Moreover, tuberculosis, at any rate in the conditions under which the vast bulk of cattle live, is but feebly infectious.

Such being the problem, let us inquire how, on the one hand, clear-headed and logical France, and how, on the other, slow-witted but shrewd and practical Denmark have set about solving it. The motto of the Frenchman in the business is "Kill, kill, kill." It is enacted by the Ministry of Agriculture that if an animal be "obviously tuberculous" it must be immediately killed. If it only present "suspicious symptoms" it must be compulsorily tested by the injection of tuberculin. A confirmatory reaction to the tuberculin test is to be followed by prompt slaughter. If an animal, though apparently healthy itself, be known to have been in cohabitation with a tuberculous animal, it must be subjected forthwith to the tuberculin injection test. If it reacts unfavourably, it must, with certain specified exceptions, be slaughtered within a year. That, in brief, is the French method; and if tuberculosis in cattle were widespread, very dangerous to human life, and impossible to be coped with in any other way, the drastic nature of the extirpatory process might be submitted to in the interests of the general public. But when it is remembered that bovine tuberculosis is not very highly dangerous to human life, is not very widespread, and is quite capable of being coped with in other ways; when, too, it is borne in mind that the French Ministry of Agriculture gives only partial compensation to those agriculturists whose animals it summarily destroys in the public interests, it is obvious that the methods of France could not be adopted in this country except on the plea of much more urgent necessity than has yet been shown.

Denmark has acted differently in this matter. She recognises its importance as much as France does; but she is not carried away by the imaginative logic of the scientific enthusiast. To the knowledge of the scientist she has added the judgment of the experienced agriculturist, who remembers that bovine tuberculosis was born before science, and that populations have lived through many ages of it with comparatively little injury to either happiness or life. Denmark says that tuberculosis in cattle is first and chiefly the affair of the owner of the cattle. She teaches the owner that so mortal a disease is dangerous to his herds and to himself, as well as to the general public; and she offers him tuberculin without payment, and the services of the veterinary surgeon without fee, in order that he may test the condition of any suspected animals he may have upon his farm. The suspected animals she advises him to isolate. If he possesses any which are beyond the stage of mere suspicion, these she advises him to kill; and that in his own interests first and chiefly. Compensation she offers him none. Denmark is eminently practical; and she is scientific too, but in her own way.

What we in England ought to do is to take measures to ensure the prompt slaughtering of all cattle which are "obviously tuberculous"; and the most certain means of effecting this object would be to offer reasonable compensation for every animal thus killed in the interests of the public. That done there would only remain the discovery and isolation of those apparently healthy animals which are really in the early stage of tuberculosis. The county councils might very well provide tuberculin for this purpose. What we are desirous of showing in this article is that bovine tuberculosis is a real danger to the community; that though it is not a danger which justifies panic, it is yet so important as to demand legislative action; and that such action, if set about with knowledge and experience, may be very effective without being either very costly to the State, or seriously injurious to the owners of stock in our agriculturally depressed country.

